# Plasma nevirapine levels, adverse events and efficacy of antiretroviral therapy among HIV-infected patients concurrently receiving nevirapine-based antiretroviral therapy and fluconazole

**DOI:** 10.1186/1471-2334-7-14

**Published:** 2007-03-12

**Authors:** Weerawat Manosuthi, Chatiya Athichathanabadi, Sumonmal Uttayamakul, Thanongsri Phoorisri, Somnuek Sungkanuparph

**Affiliations:** 1 Bamrasnaradura Infectious Diseases Institute, Ministry of Public Health, Nonthaburi, Thailand; 2 Faculty of Medicine Ramathibodi Hospital, Mahidol University, Bangkok, Thailand

## Abstract

**Background:**

The clinical data of plasma NVP level, safety and efficacy of antiretroviral therapy (ART) for the concurrent use of nevirapine (NVP)-based ART and fluconazole (FLU) is scanty.

**Methods:**

A retrospective study was conducted in patients who were initiated NVP-based ART between October 2004 and November 2005. The objectives were to compare NVP levels, adverse events, and 36-week efficacy of NVP-based ART between patients who did not receive FLU (group A) and those who received FLU 200 mg/day or 400 mg/day (group B).

**Results:**

There were 122 patients with mean ± SD age of 36 ± 9 years; 81 in group A and 41 in group B. Median (IQR) baseline CD4 cell count was 29 (8–79) cell/mm^3 ^in group A and 19 (8–33) cell/mm^3 ^in group B (*P *= 0.102). Baseline characteristics between the two groups were similar. Mean ± SD NVP levels were 6.5 ± 3.0 mg/L in group A and 11.4 ± 6.1 mg/L in group B(*P *< 0.001). One (2.4%) patient in group B developed clinical hepatitis (*P *= 0.336). Six (7.4%) patients in group A developed NVP-related skin rashes (*P *= 0.096). There were no differences in term of 36-week antiviral efficacy between the two groups (*P *> 0.05).

**Conclusion:**

Co-administration of NVP and daily dosage of FLU (200 mg/day and 400 mg/day) results in markedly increased trough plasma NVP level when compared to the administration of NVP alone. The concurrent use of NVP and FLU in very advanced HIV-infected patients is well-tolerated. The immunological and virological responses are favorable.

## Background

Cryptococcal meningitis has been a leading cause of mortality and morbidity among AIDS patients particularly in developing countries [1]. Before the era of combined active antiretroviral therapy (ART), approximately 5%–8% of HIV-infected patients in developed countries acquired cryptococcosis [2]. The incidence has substantially declined after the era of combined effective ART. However, it is still a major problem in developing countries [3-5].

Cryptococcal meningitis is a major opportunistic infection in HIV-infected patients, especially those who had CD4 count of less than 100 cells/mm^3 ^[4,6]. Current guidelines recommend the use of high dose of amphotericin B 0.7–1.0 mg/kg/day combined with flucytosine 100 mg/kg/day during the first two weeks of treatment. After successful induction therapy, amphotericin B and flucytosine can be discontinued and consolidation therapy with fluconazole (FLU) 400 mg/day for a minimum of eight weeks is recommended [7]. After completion of 10-week treatment, FLU 200 mg/day has been used for standard secondary prophylaxis until immune reconstitution occurs as a consequence of ART [7-10]. In addition, FLU is also the preferred azole drug for systemic treatment of candidiasis and coccidioidomycosis [11].

Regarding ART, nevirapine (NVP)-based ART is an alternative non-nucleoside reverse transcriptase inhibitor (NNRTI)-based ART [12,13]. The potential adverse events of NVP including skin rashes and hepatotoxicity that generally occur within the first six weeks are well described [14,15]. The Thai Government Pharmaceutical Organization has produced a fixed-dose combination of stavudine (d4T) 30 or 40 mg, lamivudine (3TC) 150 mg, and NVP 200 mg (GPO-VIR), which has been available on the market since 2002. This new combination formula makes simple dosing feasible by taking one tablet twice daily. The previous study of bio-equivalence showed that NVP concentrations were within international and manufacturer's standard [16]. The majority of HIV-infected patients in Thailand have received this combined pill due to more affordable cost [17-19].

Nonetheless, the potential drug-drug interaction between NVP and FLU is a major concern. NVP induces cytochrome P450 isoenzymes in the liver while FLU inhibit the activity of this enzyme [20-22]. A previous report has demonstrated that fluconazole significantly raises plasma NVP levels and may cause serious hepatotoxicity [23]. Conversely, NVP does not significantly influence the plasma level of FLU. However, there have not been enough data or any recommendations to adjust NVP dosage for the concurrent use of both drugs in order to avoid the adverse events. There is little clinical data of safety and tolerability for concurrent use of NVP and FLU. We therefore conducted this retrospective study to compare the trough plasma NVP levels and frequencies of adverse events among antiretroviral HIV-infected patients who did not receive FLU and received FLU for cryptococcosis prophylaxis or treatment and concurrently received NVP-based ART regimens.

## Methods

A retrospective study was conducted among antiretroviral-naïve HIV-infected patients who were initiated NVP-based ART with a fixed-dose combination of d4T, 3TC and NVP 200 mg (GPO-VIR) between October 2004 and November 2005 at Bamrasnaradura Infectious Diseases Institute, Ministry of Public Health, Nonthaburi, Thailand. The clinical data were retrospectively reviewed and retrieved from case records. Inclusion criteria were as follows: (1) HIV-infected patients >15 years of age, (2) naïve to antiretroviral therapy, (3) were initiated with a NVP-based ART regimen, (4) used NVP 200-mg once-daily lead-in dose during the first 2 weeks, prior to escalation to 200 mg twice daily i.e. patients received a fixed-dose combination tablet in the morning time and then received separate tablet of d4T and 3TC in the evening at 12 hours apart.

The patients were excluded if baseline creatinine level was higher than 2.0 mg/ml; baseline liver aminotransferase enzyme was higher than five times of upper normal limit, or receiving a medication that has drug-drug interactions with NVP or FLU, or FLU dosage was changed during the first six weeks of antiretroviral treatment. Each eligible patient was categorized into one of the two groups according to whether the patient received FLU or not as follows: did not receive FLU (group A) or received FLU 200 mg/day or 400 mg/day (group B). The trough plasma NVP levels were performed after six weeks of NVP-based ART. All patients in group B did not have the dosages of FLU changed before measurement of NVP level. Liver enzymes were monitored at 12 weeks of ART and when patients developed clinical signs and symptoms suggested for clinical hepatitis.

Regarding treatment of cryptococcosis, patients received an 8-week induction of FLU 400 mg/day after 2-week amphotericin B; and followed with FLU 200 mg/day for maintenance treatment. The patients in group B who were initiated NVP-based ART while receiving 400 mg/day had trough plasma NVP levels performed before FLU dose reduction. The others who were initiated NVP-ART while receiving FLU 200 mg/day had received both drug through the end of the study. All patients were followed for 36 weeks after initiating NVP-based ART.

The primary objective was to compare the mean trough plasma NVP levels between the two groups. The secondary objectives of interest were as follows: 1) to compare the frequencies of clinical hepatitis, skin rashes and elevated liver function test including alkaline phosphatase (ALP), aspatate aminotransferase (AST), alanine aminotransferase (ALT) and total bilirubin at three months after initiation of NVP-based ART and 2) to compare immunological and virological response between the two groups.

Blood samples for NVP level were obtained 12 hours (by patients observed taking dosing) after drug administration to analyze NVP levels at the HIV Netherlands-Australia-Thailand (HIV-NAT) Research Pharmacokinetic Laboratory located at Chulalongkorn Medical Research Center (Chula MRC) by high performance liquid chromatography (HPLC) assay. The assay was developed in Department of Clinical Pharmacology at the University Medical Centre Nijmegen, the Netherlands. The HPLC system consisted of a model P4000 solvent delivery pump, a model AS3000 autosampler, a model UV2000 programmable UV detector wavelength 251 nm and the computing integrator for HPLC model SN4000 system. The units were all from Thermo Finnigan (San Jose, California, U.S.A.). The analytic column was an Omnisher 5 C18 column (150 × 4.6 mm ID; particle size, 5 μm) protected by a Chromguard RP column – both of which were from Varian (Middelburg, The Netherlands). Analytic column runs were processed by Millennium32 software from Water (Etten-Luer, The Netherlands). The NVP retention time was 1.8 minutes. The NVP calibration curve was linear over a range of 0.055 to 15.700 mg/l. The lower limit of quantification for NVP was 0.05 mg/l. Recovery after extraction from plasma was 101.8 ± 4.6%. Accuracy in plasma ranged from 102% at 0.083 mg/L, 101% at 0.829 mg/l and 103% at 4.149 mg/l (n = 15). Within-day precisions ranged from 6.40% at 0.083 mg/l, 4.97% AT 0.830 mg/l and 2.69% at 4.149 mg/l. Between-day precisions ranged from 3.65% at 0.083 mg/l, 0% at 0.830 mg/L and 0% at 4.149 mg/l.

Mean (± standard deviation, SD), median (interquartile range, IQR) and frequencies (%) were used to describe patients' characteristics in each treatment groups. Chi-square test and Mann-Whitney test were used to compare categorical and continuous variables respectively between the two treatment groups. Paired T-test was used to compare liver function test between baseline and three months after initiating NVP. All analyses were performed using SPSS program version 11.5. A *P *value less than 0.05 was considered statistically significant. The study was approved by the ethical review board of the institute.

## Results

There were 199 patients who were initiated NVP-based ART during the period of study. Among these patients, 72 cases and 5 cases were excluded from the study due to receiving medications that have drug-drug interactions and having high baseline liver transaminase enzymes, respectively. A total of 122 patients with a mean ± SD age of 36 ± 9 years and 55% male were eligible and included in the study. Of 122 patients, 81 and 41 patients were classified into group A and B, respectively. The patients' baseline characteristics between the two groups are shown in Table 1. Baseline characteristics including age, gender, body weight, serum ALP, AST, ALT, total bilirubin, CD4 cell counts and plasma HIV RNA between the two groups were not different. All patients received the same backbone antiretroviral drugs including stavudine and lamivudine.

**Table 1:** Baseline characteristics between the two study groups

Demographics	Group A *(n = 81)*	Group B *(n = 41)*	*p *value
Age, years, mean ± SD	37 ± 9	35 ± 8	0.310
Male gender	44 (54.3%)	23 (56.1%)	1.000
Body weight, kgs, mean ± SD	54.1 ± 10.3	50.8 ± 9.9	0.095
Baseline CD4 counts, cell/μl, median (IQR)	29 (8–79)	19 (8–33)	0.102
Baseline plasma HIV RNA, copies/ml, median (IQR)	271,500 (95,353–714,000)	201,000 (73,125–688,500)	0.269
Baseline ALP, U/l, median (IQR)	85.5 (65.8–126.3)	93.0 (66.0–141.0)	0.565
Baseline AST, U/l, median (IQR)	34.0 (25.3–47.8)	34.0 (21.0–54.0)	0.636
Baseline ALT, U/l, median (IQR)	32.5 (22.0–48.3)	28.5 (13.8–52.3)	0.225
Baseline total bilirubin, mg/dl, median (IQR)	0.5 (0.4–0.7)	0.6 (0.3–0.8)	0.561

ALP = alkaline phosphatase, AST = aspatate aminotransferase, ALT = alanine aminotransferase

**Figure 1:**
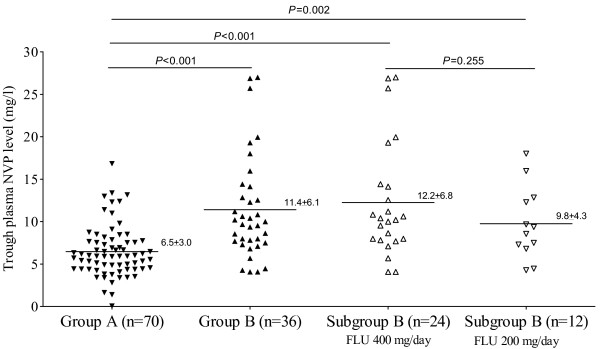
The distribution of trough plasma NVP levels among the study groups.

The distributions of trough plasma NVP levels are shown in Figure 1. Mean ± SD NVP levels were 6.5 ± 3.0 and 11.4 ± 6.1 mg/l in group A and B, respectively (*P *< 0.001). Of 41 patients in group B, 14 patients were receiving FLU 200 mg/day and 27 patients were receiving FLU 400 mg/day when NVP levels were performed. Mean trough NVP level was 9.8 ± 4.3 mg/l in the patients receiving FLU 200 mg/day and 12.2 ± 6.8 mg/l in the patients receiving FLU 400 mg/day (P = 0.255). Mean trough plasma NVP level in group A was also significantly lower than that in each subset of group B patients (*P *< 0.001).

Seventy patients in group A (6 [7.4%] rash, 3 [3.6%] died, 2 [2.4 %] lost to follow-up) and 33 patients in group B (4 [9.7%] died, 3 [7.3%] lost to follow-up, 1 [2.4%] hepatitis) continue to follow-up until 36 weeks of ART. There was a higher tendency of drug discontinuation from all causes in group B (*P *= 0.073). The causes of death among 7 patients were not related to the adverse events. All causes of death were related to opportunistic infections or HIV infection, including cerebral toxoplasmosis, *Mycobacterium avium *complex infection, CMV radiculomyelitis, sepsis and wasting syndrome.

CD4 cell count and plasma HIV RNA were not performed in 7 patients (3 in group A and 4 patients in group B). Therefore, 67 patients in group A and 29 patients in group B were evaluated for virological and immunological response by on-treatment analysis. After 36 weeks of ART, 61 of 67 (91.04%) patients in group A and 26 of 29 (89.66%) in group B achieved undetectable plasma HIV RNA (*P *= 0.549). Group A patients had median CD4 cell counts of 104, 152 and 192 cells/mm^3 ^at week 12, 24 and 36, respectively. Group B patients had median CD4 cell counts of 94, 119 and 161 cells/mm^3 ^at the corresponding periods. There was no difference of CD4 change from week 0 to week 36 between the two groups (*P *= 0.869). Median (IQR) CD4 cell count changes at 36 weeks from baseline were 155 ± 95 cells/mm^3 ^in group A and 152 ± 134 cells/mm^3 ^in group B (*P *= 0.869).

Liver enzymes at 12 weeks of ART between the two groups are shown in Table 2. The patients in group A had higher level of serum ALP than the patients in group B (P = 0.011). There were no differences of AST, ALT and total bilirubin between the two groups (*P *> 0.05). By repeated measurement analysis, ALP and ALT levels after 12 weeks of NVP-based ART were not different from baseline in both groups (*P *> 0.05). The frequencies of adverse events including clinical hepatitis and skin rashes are shown in Table 2. Six of 81 (7.4%) patients in group A developed skin rashes in which all events occurred within the first 6 weeks of ART; one of 41 (2.4%) patients in group B developed clinical hepatitis after 5 weeks of ART. Among the patients who experienced cutaneous reactions, all patients developed diffuse erythematous maculopapular rashes grade II and grade III. No patient developed severe skin reactions such as Stevens Johnson syndrome or toxic epidermal necrolysis. ART in all patients with adverse events was switched to efavirenz-based ART and they could tolerate well.

**Table 2:** Liver enzymes and adverse events after 12 weeks of ART

Parameters	Group A (n = 81)	Group B (n = 41)	*p *value
*Liver enzymes*			
ALP at week 12, U/l, median (IQR)	81.5 (65.0–116.8)	77.0 (112.0–132.5)	0.011
AST at week 12, U/l, median (IQR)	20.3 (25.0–33.8)	22.0 (27.5–44.3)	0.351
ALT at week 12, U/l, median (IQR)	17.0 (25.0–38.8)	22.0 (26.0–46.0)	0.329
TB at week 12, mg/dl, median (IQR)	0.3 (0.4–0.5)	0.3 (0.4–0.5)	0.907
*Adverse events*			
Skin rashes	6 (7.4%)	0 (0%)	0.096
Clinical hepatitis	0 (0%)*	1 (2.4%)	0.336

* add 0.5 to calculate *p *value

A 31-year old male in group B who developed clinical hepatitis had a baseline CD4 cell count of 17 cells/mm^3 ^and plasma NVP level of 10.2 mg/l. He developed symptoms of nausea and vomiting after five weeks of NVP. Liver function test showed combined cholestasis and hepatotoxicity. The other potential causes of hepatitis were excluded. Serology of hepatitis B virus and hepatitis C virus were negative. NVP, d4T and 3TC were discontinued; clinical symptoms and liver function test results turn to be normal within three weeks. d4T, lamivudine and efavirenz were then prescribed. He could tolerate this second regimen well.

## Discussions and conclusion

In the present study, we demonstrate that co-administration of NVP and daily dosage of FLU (200 mg/day and 400 mg/day) results in markedly increased trough plasma NVP level when compared to the administration of NVP alone. From the MEDLINE database, the present study is the first study to date documenting plasma NVP levels among advanced HIV-infected patients who concurrently received NVP and FLU versus those who did not received FLU.

As we know that cryptococcosis occurs in advanced HIV-infected patients who are vulnerable to other opportunistic infections. ART should not be delayed when cryptococcal diseases are improved. NVP-based ART is most affordable ART regimen in resource-limiting countries. Some physicians may somewhat reluctant to initiate NVP-based ART while the patients are receiving FLU for treatment or prophylaxis of cryptococcosis. Deference of ART may increase the risk of disease progression and intervening opportunistic infections. From these reasons, we attempted to determine the plasma NVP levels and adverse events while the patients were concurrently receiving FLU.

The plasma half-life of either FLU or NVP are approximately 30 hours after oral administration [11]. The steady state of metabolism of both drugs had been achieved when plasma NVP levels were measured. The timing period of exposure to both NVP and FLU in all patients in group B was at least six weeks. This period is enough to evaluate not only steady state of plasma NVP levels but also the frequency of adverse events. The results show that plasma NVP levels among patients in group B (11.6 mg/l) were approximately 76% higher than the plasma NVP level in group A (6.6 mg/l). Currently, the recommended optimal trough plasma NVP levels should be higher than 3.4 mg/l [13]. Our data corresponds to the previous study that conducted in stable African HIV-infected patients on ART with an average CD4 cell count of 375 cell/mm^3 ^who received FLU 200 mg/day [23]. Seventy percents of patients in this previous study were female. Herein, we demonstrate the actual situation of common concomitant use of NVP and FLU in advanced HIV-infected patients. Geel and colleagues demonstrated that trough plasma NVP level was 9.4 mg/l in patients concurrently received NVP and FLU 200 mg/day. However, the plasma NVP level in our patients who received FLU 200 mg/day was comparable to the result from this previous study [23]. In addition, the present study also demonstrated that mean trough plasma NVP levels were not different between patients receiving daily 200 and 400 mg. One reason that may explain this could be that FLU 200 mg/day maximally inhibit enzyme that metabolizes NVP. Thus, drug-drug interaction between NVP and FLU may have a ceiling effect. However, the power of sample size may not enough to detect this difference.

Liver toxicity is an important complication among HIV-infected patients who receive NVP-based ART. This drug can increase potential for liver toxicity through either hypersensitivity reaction during the early period of ART or dose-dependent effect [24]. The results from the present study showed that there was no difference of clinical hepatitis or elevated liver function test between the two groups. This finding corresponds to a previous large retrospective study which demonstrated the safety of co-administration of NVP and FLU [25]. However, one patient (2.4%) in group B developed clinical hepatitis at five weeks after NVP-based ART. Both NVP and FLU are potential causes of hepatitis. However, there have been rare cases of serious hepatic reactions from FLU [26,27]. In this patient, only NVP-based ART was discontinued and then the liver function test had returned to be normal within three weeks. This may presume that the clinical hepatitis in this patient was caused by NVP.

A previous study indicated that when the higher plasma NVP level is achieved, the higher frequency of adverse events occurred [28]. However, a more recent study demonstrated that plasma NVP level did not have a relationship to adverse events when corrected for known covariates [29]. The value of periodical drug monitoring of NVP as a way to prevent toxicity is therefore limited. Physicians should instead focus on factors that are more predictive of adverse events including gender, CD4 cell count and hepatitis coinfection. Geel and colleagues reported that 8.3% and 16.7% of patients who concurrently received NVP and FLU developed clinical hepatitis and elevated aminotransferase enzymes [23]. The prevalence of clinical hepatitis in the present study is markedly low when compare to this previous report. This discrepancy can be explained by the lower number of CD4 cell count in the present study and different proportion of gender. Almost all of patients had CD4 cell counts of less than 100 cells/mm^3^. In a published analysis of NVP-associated hepatotoxicity, sex-dependent CD4 cell count was found to be predictive of hepatitis [30]. High CD4 cell count is a predictor of hepatotoxicity from NVP [14]. Thus, concomitant administration of NVP and FLU shall not be contraindicated especially among co-infected HIV and cryptococcus patients since these patients usually have very low CD4 cell count.

Regarding NVP-associated skin rashes, there was a trend toward higher prevalence of skin rashes in the patients who received NVP-based ART alone than another group. This may be explained by the random variation of the study. Notably, although the patients in group B had significantly higher plasma NVP levels, they had a low rate of skin rashes. Thus, NVP level is not a predictive factor for this adverse event that correspond to the previous study [29]. To date, the mechanism of NVP-associated skin rashes is still not clear. The patients in group B had a higher tendency of dead and lost to follow-up but there were no differences between the two groups. These may be partly explained by that the patients in group B had more advanced disease.

Regarding antiviral efficacy, there was no difference in term of virological outcome between the two treatment groups after 36 weeks of ART (*P *= 0.549). In addition, CD4 cell counts change at week 36 from baseline value was not different (*P *= 0.869), CD4 cell count at week 36 was significantly higher than at baseline value (*P *< 0.001), Therefore, NVP-based ART showed effective antiviral efficacy among very advanced HIV-infected patients in both study groups. This result corresponds to the previous studies that conducted in Thais [17-19].

NVP-based ART is a common regimen which is widely used for the treatment of HIV-infected patients in resource-limited countries. Until the other options are more accessible, NVP-based ART is still a key regimen to scale up treatment of HIV in resource-limited countries. The fact remains that many settings in these countries are still unable to avoid NVP-based ART in advanced co-infected HIV and cryptococcosis patients. Thus, the examining of drug-drugs interaction between NVP-based ART and other drugs widely used in HIV-infected patients is crucial. In addition to the treatment and prophylaxis of cryptococcosis, FLU is the preferred azole drug for systemic treatment other common opportunistic infections in HIV-infected patients, such as esophageal candidiasis, coccidioidomycosis and alternative treatment of histoplasmosis [11]. Our study may provide the safety data of concomitant use of NVP and FLU for caring HIV-infected patients.

Some important pharmacokinetic and clinical data were not evaluated and some selection bias may be occurred due to the nature of retrospective study. First, plasma NVP levels were not performed between the first few weeks of ART while NVP level was not in the steady state. The frequencies of NVP-associated adverse events were at peak during this period. Second, NVP levels were not performed among the patients who needed to discontinue NVP during the first few weeks of ART. Third, the proportion of patients who received NVP and daily dose FLU may not be large enough to detect the difference of relatively low incidence of adverse events and the difference of plasma NVP levels between the patients who received FLU 200 mg/day and FLU 400 mg/day. However, our previous large retrospective study indicated that there was no difference of clinical hepatitis between patients who received NVP with FLU 200 mg/day and NVP alone [25]. Forth, the correlation of genetic deposition and plasma NVP levels was not studied. Fifth, plasma FLU level was not evaluated. However, NVP does not significantly influence the plasma level of FLU as mentioned. Sixth, some patients lost to follow-up and died due to superimposed opportunistic infections during the study period. Thus, the final outcomes of these patients are not established. Lastly, the data may not be applicable for the patient who has high CD4 cell count and the other ethnic populations.

In conclusion, the frequencies of adverse events during the early period of concurrent use of NVP and FLU were comparable with use of NVP without FLU, in spite of significantly higher plasma NVP levels at six weeks of ART. The standard dose of NVP should be safe among advanced HIV-infected patients receiving FLU. The 36-week virological and immunological responses are not different between the two groups. The concurrent administration of NVP and daily dose of FLU in advanced HIV-infected patient is well-tolerated and efficacious.

## Abbreviations

ART: active antiretroviral therapy, FLU: fluconazole, NVP: nevirapine, ALP: alkaline phosphatase, AST: aspatate aminotransferase, ALT: alanine aminotransferase

## Competing interests

The author(s) declare that they have no competing interests.

## Authors' contributions

WM participated in the design of the study, performed statistical analysis and draft the manuscript. CA participated in the design of the study. SU participated in the design of the study. TP participated in the design of the study. SS participated in the design of the study and helped to draft the manuscript. All authors read and approved the final manuscript.

## Pre-publication history

The pre-publication history for this paper can be accessed here:


